# Glucose‐Responsive PEGDA‐GelMA‐MPBA Hydrogel Loaded with Exosomes Promotes Diabetic Wound Healing

**DOI:** 10.1155/jdr/3449648

**Published:** 2026-05-06

**Authors:** Weiyan Yuan, Qiuru Zhuang, Qian Liu, Zhier Lin, Jiaqi Bu, Yumin Zhuo, Yijing Zhou, Xiaoying Yan, Jing Zheng

**Affiliations:** ^1^ School of Nursing, Guangdong Pharmaceutical University, Guangzhou, Guangdong, China, gdpu.edu.cn

**Keywords:** diabetic wound healing, exosomes, glucose-responsive, immunomodulation, PEGDA-GelMA-MPBA hydrogel

## Abstract

Diabetic wound healing is frequently impaired by microcirculatory deficiencies and a proinflammatory microenvironment under conditions of persistent hyperglycemia. This study presents a smart multifunctional hydrogel dressing based on a ternary‐network system of poly(ethylene glycol) diacrylate (PEGDA), gelatin methacryloyl (GelMA), and 3‐methacrylamidophenylboronic acid (MPBA) for sustained delivery of exosomes. The resulting PEGDA‐GelMA‐MPBA@Exos (P‐G‐M@Exos) hydrogel exhibits high hydrophilicity, tunable mechanical strength, potent antibacterial activity, and favorable biocompatibility. A key innovation of this system is its glucose‐mediated exosome release through dynamic boronate ester bonds, enabling on demand delivery of bioactive molecules in response to hyperglycemic conditions. In vivo, the hydrogel significantly enhanced M2 macrophage polarization, mitigated excessive inflammation, and promoted functional angiogenesis by upregulating *VEGF*. These coordinated action ultimately facilitated rapid wound closure, enhanced collagen remodeling, and promoted re‐epithelialization. Our study offers a promising therapeutic platform for intelligent wound management, demonstrating strong translational potential for diabetic wound care.

## 1. Introduction

Diabetic foot ulcers (DFUs) affect up to 34% of diabetic individuals over their lifetime, with approximately 18.6 million new cases reported globally each year [1]. DFUs are responsible for approximately 80% of nontraumatic lower limb amputations in this population [2]. These wounds are chronic and refractory, primarily due to diabetic vasculoneuropathy and recurrent bacterial infections. The DFU microenvironment is complex, marked by chronic hyperglycemia, ischemia, hypoxia, excessive inflammation, and a high risk of infection [3, 4]. Current clinical management of DFUs remains suboptimal. Standard care typically involves advanced dressings, including hydrogels, foam, alginate, and antimicrobial‐based formulations. Although such materials help maintain a moist wound environment and provide limited anti‐infective benefits, they fall short in promoting substantial tissue regeneration and facilitating controlled release of bioactive molecules [5]. Current treatments do not meet the complex biological needs of diabetic wound healing, underscoring the critical need for innovative therapeutic approaches.

The combination of nanotechnology and bioactive carriers (e.g., solid lipid nanoparticles, exosomes, phytosomes) has opened new avenues for improving drug targeting and stability in diabetic wound therapy [6–8]. However, developing a cohesive platform that mimics the extracellular matrix, adapts to the dynamic wound microenvironment, and integrates multiple therapeutic mechanisms remains a considerable challenge. Hydrogels have attracted considerable interest as wound dressing materials due to their excellent moisture retention and favorable biocompatibility [8, 9]. Their three‐dimensional porous network structure provides an ideal moist environment and serves as an effective platform for the controlled release of bioactive agents, including growth factors, stem cells, and exosomes [10, 11]. Among these, exosomes—particularly those derived from human umbilical cord mesenchymal stem cells (hUC‐MSCs) have gained attention for their enhanced proregenerative capabilities compared with bone marrow‐derived exosomes, as well as their noninvasive isolation and minimal ethical concerns [12–14]. However, the translational potential of exosomes is limited by their rapid systemic clearance and brief half‐life, which restricts their local retention and prolonged bioactivity within the wound bed [15, 16].

PEGDA, a synthetic polymer widely used in biomedical applications, offers excellent biocompatibility and tunable mechanical properties [17]. Its UV‐initiated crosslinking capability makes it ideal for creating drug delivery systems and tissue engineering scaffolds [18, 19]. To enhance its biological functionality, we incorporated GelMA as a key component in this study. GelMA possesses high biocompatibility, adjustable mechanical properties, and contains cell‐interactive motifs that support the efficient loading of bioactive components (e.g., exosomes) and facilitate favorable cell‐material interactions. Its efficacy has been demonstrated in multiple diabetic wound healing models [20–22]. Despite these advantages, conventional PEGDA‐GelMA systems lack inherent antibacterial properties and glucose‐responsive behavior, limiting their effectiveness in the complex diabetic wound milieu.

Consequently, creating smart wound dressings with glucose‐responsive properties has emerged as a pivotal research direction [8, 23]. MPBA can undergo dynamic boronate ester formation with glucose, enabling competitive binding and dissociation that facilitates glucose‐dependent drug release. MPBA has shown promise in pioneering glucose‐sensitive systems [24, 25] and also exhibits intrinsic antibacterial properties that may help mitigate wound infection risks [26]. Although MPBA has been explored in glucose‐responsive materials, its integration with PEGDA and GelMA via photo‐crosslinking to construct a novel ternary‐network hydrogel capable of spatiotemporal exosome release remains unreported. This study is aimed at developing a mechanically robust, antibacterial, and glucose‐responsive P‐G‐M ternary‐network hydrogel and evaluate its synergistic effects on immunomodulation and tissue regeneration in diabetic wounds following exosome incorporation.

## 2. Materials and Methods

### 2.1. Culture of hUC‐MSCs and Isolation/Characterization of Exosomes

The supernatant obtained from hUC‐MSC cultures was acquired from Engineering For Life Co. Ltd. The isolation of hUC‐MSCs was performed according to an established protocol [27], as detailed in a previous publication [28]. The supernatant was centrifuged sequentially at 300 × g and 2000 × g for 10 min each at 4°C to eliminate cells and debris, followed by an additional centrifugation at 10,000 × g for 30 min at 4°C. The supernatant underwent two additional processing steps, culminating in centrifugation at 100,000 × g for 70 min at 4°C. The pelleted exosomes were resuspended in 500 *μ*L of phosphate‐buffered saline (PBS) and kept at −80°C. Transmission electron microscopy (TEM) was used for morphological examination, nanoparticle tracking analysis (NTA) measured size distribution, and Western blotting identified specific exosomal markers TSG101, CD81, and CD63.

### 2.2. Preparation of P‐G‐M Hydrogel

A GelMA stock solution with a concentration of 10% (*w*/*v*) was created by dissolving 1 g of GelMA powder (EFL, China) in 10 mL of PBS with magnetic stirring at 37°C for 1 h. The solution was filtered sterilely using a 0.22‐*μ*m membrane (Millipore, United States) and kept at 4°C. MPBA (Bidepharm, China) was dissolved in a dimethyl sulfoxide (DMSO)/water mixture as described previously [24]. A mixture was prepared using PEGDA (Mn = 1 kDa; Sigma‐Aldrich, United States), GelMA stock solution, MPBA solution, and the photoinitiator lithium phenyl‐2,4,6‐trimethylbenzoylphosphinate (LAP; EFL, China) at an optimal predetermined ratio. Exposure to 405 nm UV light for 30 s initiated covalent crosslinking, incorporating MPBA into the PEGDA‐GelMA network to create a glucose‐responsive ternary hydrogel, termed P‐G‐M. Exosomes were added to the precursor solution prior to photo‐crosslinking, enabling their encapsulation within the forming three‐dimensional hydrogel matrix. To guarantee safety by eliminating potentially harmful residues, the resulting hydrogels were soaked in 1× PBS for 48 h after fabrication, with the PBS being replaced every 8 h to effectively remove any unreacted monomers and the photoinitiator.

### 2.3. Mechanical Testing

The hydrogels′ mechanical characteristics were evaluated with a universal testing system. Cylindrical samples (5 mm diameter, 10 mm thickness) underwent compression at a constant rate of 5 mm/min at room temperature (RT). The compressive modulus was determined from the slope of the linear elastic portion of the stress‐strain curve.

### 2.4. Morphological Characterization

Cylindrical P‐G‐M hydrogels (1 cm diameter, 2 mm thickness) were immersed in either PBS or high‐glucose DMEM (DMEM with 4.5 g/L glucose; in subsequent experiments, “high‐glucose DMEM” refers to this composition) for 24 h, lyophilized, and then examined by scanning electron microscopy (SEM). Pore size distribution was quantified using ImageJ software (v1.8.0; NIH, United States).

### 2.5. Exosome Release and Distribution

To assess the release kinetics, cylindrical hydrogel discs, identical in dimensions to those outlined in Section 2.4, were prepared. Each disc contained 100 *μ*g of exosomes and was then incubated in 200 *μ*L of either PBS or high‐glucose DMEM. At predetermined intervals over 14 days, 20 *μ*L of supernatant was removed and replaced with the same amount of new solution. Exosome levels were measured via a BCA protein assay. Exosomes were labeled with PKH26 fluorescent dye, integrated into hydrogels at a concentration of 100 *μ*g, and examined using a microscope to visualize spatial distribution.

### 2.6. Antibacterial Assay

The evaluation of antibacterial activity was conducted using the agar plate colony counting technique. Sterile P‐G‐M hydrogels (1 cm diameter, 2 mm thickness), Duoderm dressings of identical size (positive control), and empty wells (negative control) were placed in a 24‐well plate. Each well received 100 *μ*L of bacterial suspension, either *Escherichia coli DH5α* or *methicillin-resistant Staphylococcus aureus (MRSA)*, at a concentration of approximately 10^5^ CFU/mL. After incubating for 24 h with shaking at 37°C and 250 rpm, 30 *μ*L of culture from each well was plated onto LB agar. The plates were incubated at 37°C for 24 h without agitation, after which the colony‐forming units (CFUs) were counted.

### 2.7. Cell Proliferation Assay

Cylindrical hydrogels (1 cm diameter, 2 mm thickness) were immersed in 500 *μ*L of cell culture medium at 37°C for 24 h, with a medium‐only setup as the control. Human umbilical vein endothelial cells (HUVECs) were seeded at a density of 10^6^ cells/mL in 24‐well plates (with or without hydrogels). Cells were stained with a fluorescent dye and imaged directly after 24 or 48 h of incubation. Cell proliferation was also quantified using a CCK‐8 assay kit (Glpbio, United States).

### 2.8. Cell Migration Assay

HUVECs were cultured alongside the hydrogel for 48 h in a Transwell insert, with cells in the top chamber and the bottom chamber filled with medium containing 1% FBS. After 12 h, nonmigrated cells on the upper surface were removed with a cotton swab. Migrated cells were fixed with 4% paraformaldehyde, stained using crystal violet, and examined under a Nikon Eclipse Ti inverted microscope.

### 2.9. Diabetic Mouse Wound Model

Approval for all animal procedures was granted by the Institutional Animal Ethics Committee of Guangdong Pharmaceutical University. C57BL/6 male mice, between 6 and 8 weeks old, underwent overnight fasting before receiving daily intraperitoneal injections of streptozotocin (STZ) (50 mg/kg; Nanjing Doulai, China) over five consecutive days. Diabetes was confirmed 2 weeks later by measuring fasting blood glucose levels above 16.7 mM using a glucometer (RockTech, China). A biopsy punch (RockTech) was utilized to make full‐thickness dorsal skin wounds, each 1 cm in diameter, which were then covered with Tegaderm film (3 M, China). The treatment groups received the P‐G‐M precursor solution, with or without exosomes, applied to the wound site. This solution was then crosslinked in situ using 405 nm UV light for 30 s, forming a cylindrical hydrogel with a 1 cm diameter and 2 mm thickness. Wound areas were assessed on Days 0, 3, 6, 9, and 12. Wound and surrounding tissues were harvested for subsequent analysis on the 7th and 15th days following surgery. The wound closure rate (WCR) is calculated with the formula: WCR = [(*A*_0_ − *A*_t_)/*A*_0_] × 100*%*, where *A*_0_ represents the original wound area and *A*_t_ is the area at time *t* [29].

### 2.10. Histological Analysis

On the 7th and 15th days, wound tissues along with adjacent skin were collected, fixed in 4% formaldehyde, embedded in paraffin, and cut into 4‐*μ*m sections. These sections underwent staining with hematoxylin and eosin (H&E; Servicebio, China) and Masson′s trichrome (Solarbio, China) for histological evaluation. The ratio between Collagen I and III was assessed with ImageJ software.

### 2.11. Immunofluorescence Staining

Tissue sections underwent antigen retrieval in a preheated buffer at 95°C for 10 min, followed by blocking with 2% BSA. They were then incubated overnight at 4°C with primary antibodies: *α*‐SMA (1:3000; Abcam, ab7817), iNOS (1:50; Abcam, ab3523), Arg1 (1:150; GeneTex, GTX10924), collagen I (1:300; Abcam, ab21286), and collagen III (1:400; Abcam, ab7778). Sections were incubated with secondary antibodies (1:900; Abcam) for 2 h at RT after washing. DAPI was used to counterstain the nuclei. Images were captured using a Nikon Eclipse Ti confocal microscope (Japan).

### 2.12. Real‐Time Quantitative PCR (qPCR)

TRIzol reagent (Invitrogen, United States) was used for RNA extraction from tissues, and cDNA synthesis was carried out using HI Script QRT Super Mix (Vazyme, China). qPCR was performed on a Roche LightCycler 96 system utilizing 2**×** PCR master mix from GenStar, China. The 2^(−ΔΔCt)^ method was employed to analyze relative gene expression [30], and primer sequences are detailed in Table S1. Experiments were performed in triplicate, with results presented as mean ± standard deviation (SD).

### 2.13. Statistical Analysis

Data were analyzed using a two‐sample *t*‐test, one‐way ANOVA, or Wilcoxon rank‐sum test, as appropriate. A *p* value below 0.05 was deemed statistically significant.

## 3. Results and Discussion

### 3.1. Culture of hUC‐MSCs and Isolation/Characterization of Exosomes

The culture supernatant of hUC‐MSCs was subjected to differential centrifugation to obtain exosomes. TEM imaging revealed that the isolated exosomes exhibited a characteristic cup‐shaped morphology (Figure S1A). NTA demonstrated a predominant size distribution ranging from 30 to 150 nm (Figure S1B). Furthermore, Western blot analysis verified the presence of characteristic exosomal markers, such as TSG101, CD81, and CD63 (Figure S1C), in accordance with globally recognized criteria for exosome identification [31].

### 3.2. Compressive Characteristics and Glucose‐Responsive Sustained Release Behavior of the P‐G‐M Hydrogel

**Figure 1:**
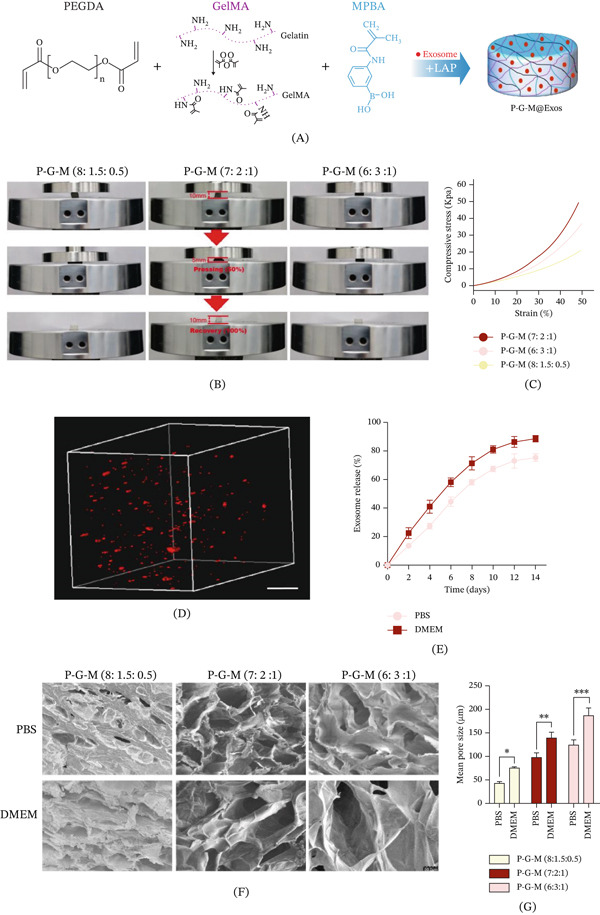
Mechanical characteristics and glucose‐responsive sustained release behavior of exosomes derived from the P‐G‐M hydrogel. (A) The P‐G‐M hydrogel is primarily composed of PEGDA, GelMA, and MPBA. (B–C) Stress‐strain profiles at a strain level of 50% for P‐G‐M hydrogels with various compositions during compression testing. (D) Three‐dimensional imaging of the P‐G‐M hydrogel integrated with PKH26 labeled exosomes, observed through fluorescence microscopy. Scale bar: 100 *μ*m. (E) Kinetics of exosome release from the P‐G‐M@Exos (7:2:1) hydrogel in either PBS or high glucose DMEM. (F–G) Scanning electron microscopy analysis illustrated the distribution and size of pores in P‐G‐M hydrogels with differing ratios following immersion in either PBS or high‐glucose DMEM. Results are presented as mean ± SD; significance levels are indicated as follows:  ^∗^ *p* < 0.05,  ^∗∗^ *p* < 0.01,  ^∗∗∗^ *p* < 0.001.

This study presents the development of a glucose‐responsive hydrogel, P‐G‐M@Exos, which is loaded with exosomes. The hydrogel was synthesized by functionalizing GelMA with MPBA, followed by crosslinking with PEGDA via photo‐initiation and subsequent encapsulation of exosomes (Figure 1A). Cyclic compression tests were conducted to assess the hydrogel′s mechanical properties. The P‐G‐M hydrogel exhibited rapid recovery to its original structure following 50% compressive strain, demonstrating excellent elastic recovery and compressive resilience (Figure 1B,C). The stress‐strain analysis of hydrogels with varying P‐G‐M ratios revealed that the P‐G‐M (7:2:1) formulation exhibited superior compressive strength, essential for preserving mechanical integrity at the wound site and reducing disruption during the healing process.

The stable encapsulation of exosomes within the hydrogel matrix is achieved primarily through physical confinement within the polymer network [12]. Uniform distribution of exosomes throughout the hydrogel was verified using PKH26 fluorescent labeling (Figure 1D). In vitro release kinetics studies demonstrated that exosomes from P‐G‐M@Exos exhibited a sustained release profile over a period of 14 days (Figure 1E), a duration that corresponds effectively with the timeline of in vivo angiogenesis processes, highlighting the system′s capability for sustained release. This three‐dimensional cross‐linked network restricts the free diffusion of exosomes, thereby reducing their susceptibility to enzymatic degradation and promoting the preservation of their biological activity and functional integrity [32].

Diabetic wound healing is frequently impaired by microcirculatory deficiencies and a proinflammatory microenvironment under conditions of persistent hyperglycemia [33]. To address this, we introduced MPBA to confer glucose responsiveness to the hydrogel. As shown in Figure 1E, the P‐G‐M@Exos hydrogel exhibited a significantly accelerated release of exosomes under high‐glucose conditions, accompanied by an increase in porosity (Figure 1F,G). The response mechanism originates from dynamic boronic ester bonds formed between the phenylboronic acid moiety in MPBA and the cis‐dihydroxy groups within the gel network. Glucose competitively binds to these bonds, inducing their cleavage and consequently causing network swelling and pore enlargement, which drives the release of exosomes [34]. It is noteworthy that this dynamic covalent bond is inherently reversible; theoretically, a decrease in glucose concentration could induce boronate ester reformation and network contraction, subsequently slowing the release [35, 36]. This potential bidirectional responsiveness to fluctuating glucose levels, however, warrants further validation in subsequent studies involving alternating glucose concentration experiments.

The constructed P‐G‐M ternary network synergistically integrates structural stability, bioactivity, and smart drug release capability, offering a novel strategy for diabetic wound management.

### 3.3. Antibacterial Activity and Biocompatibility of the P‐G‐M Multifunctional Hydrogel

**Figure 2:**
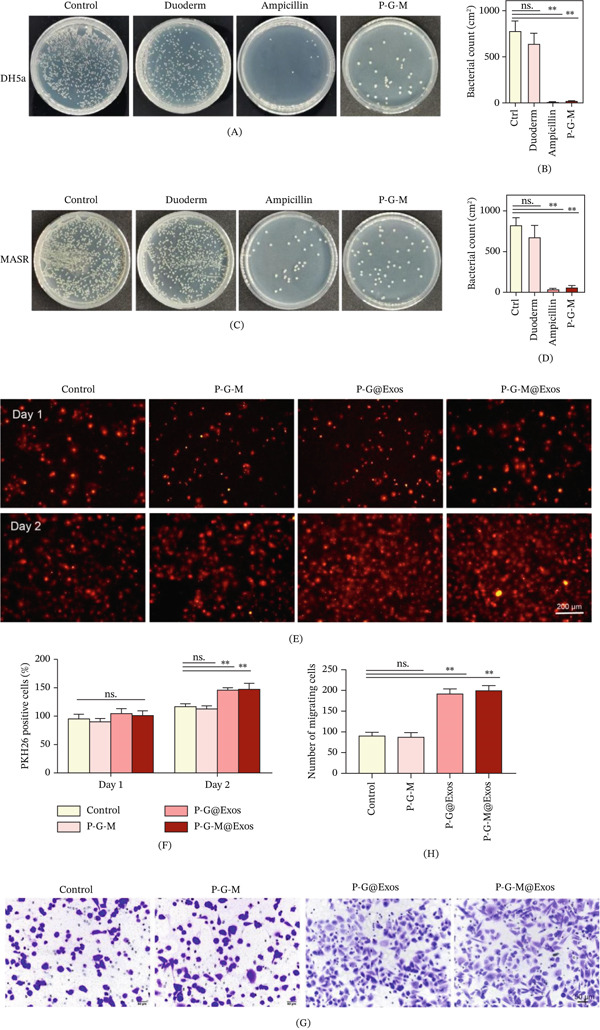
The P‐G‐M hydrogel′s antibacterial effectiveness and biocompatibility are evaluated. (A, B) *DH5a* bacterial colonies were cocultured with (A) various hydrogels for 24 h at 37°C, followed by (B) quantitative analysis, with results showing no significant differences (ns).  ^∗∗^ *p* < 0.01. (C, D) Colonies of *MRSA* under (C) identical culture conditions, with (D) quantitative results, ns: not significant.  ^∗∗^ *p* < 0.01. (E, F) HUVECs stained with PKH26 were cultured for 1 and 2 days in media from pure medium (Control), P‐G‐M, P‐G@Exos, and (E) P‐G‐M@Exos hydrogels, and the (F) fluorescence intensity was quantified. Scale bar: 200 *μ*m; ns: not significant;  ^∗∗^ *p* < 0.01. (G, H) Transwell migration assay of HUVECs after (G) 24 h of culture, along with (H) quantitative analysis, scale bar 50 *μ*m; results indicate no significance (ns) unless otherwise noted ( ^∗∗^ *p* < 0.01).

Effective prevention of wound infection remains a critical challenge in wound management, particularly in diabetic patients due to their increased susceptibility. Bacterial infections can provoke neutrophils and macrophages to release substantial quantities of proinflammatory mediators [37]. Hence, antibacterial efficacy is a vital criterion in the evaluation of advanced wound dressings. The antibacterial efficacy of the P‐G‐M hydrogel was assessed against *DH5α* and *MRSA*. The results demonstrated strong antibacterial efficacy against both pathogens, with inhibitory effects comparable with amoxicillin and superior to the conventional Duoderm dressing (Figure 2A–D). This antimicrobial activity is largely attributable to MPBA, as phenylboronic acid groups can disrupt bacterial cell wall integrity at specific concentrations while maintaining low cytotoxicity toward mammalian cells [24].

Biocompatibility is another essential attribute for wound dressing materials [33]. The effect of the P‐G‐M hydrogel on cellular viability postexosome release was assessed by culturing HUVECs with hydrogel extracts for 24 and 48 h, followed by PKH26 staining and CCK‐8 assays. After 24 h, all hydrogel‐treated groups showed similar viability to the control, demonstrating excellent cytocompatibility. By 48 h, the exosome‐loaded groups (P‐G@Exos and P‐G‐M@Exos) significantly promoted HUVEC proliferation (Figure 2E,F).

Cell migration assays demonstrated that the exosome‐loaded groups significantly increased HUVEC migration compared with the control (Figure 2G,H). Collectively, these findings suggest that P‐G‐M@Exos facilitates angiogenic activation via controlled exosome release, thereby enhancing the proliferation, migration, and angiogenic potential of HUVECs.

In summary, the P‐G‐M hydrogel exhibits high hydrophilicity, potent antibacterial properties, excellent biocompatibility, and importantly, glucose‐regulated sustained exosome release. These multifunctional characteristics, combined with its glucose‐responsive release kinetics, position it as a highly promising and clinically relevant candidate for advanced diabetic wound dressings.

### 3.4. P‐G‐M@Exos Hydrogel Promotes Diabetic Wound Healing

A STZ‐induced diabetic mouse model was used to assess the therapeutic efficacy of the P‐G‐M@Exos hydrogel in diabetic wound repair (Figure S2A). The study found that diabetic mice exhibited significantly delayed wound healing, with wounds remaining unhealed by Day 20, in contrast to normoglycemic mice, whose wounds were nearly fully re‐epithelialized by Day 12 (Figure S2B). Research indicates that subcutaneously injected exosomes are quickly eliminated, becoming almost undetectable within 24 h. In contrast, exosomes delivered through PEI‐modified electrospun fibers persist for up to 7 days [38]. The short lifespan and rapid clearance of free exosomes greatly restrict their therapeutic potential. Notably, our in vitro release studies demonstrated that the P‐G‐M hydrogel facilitated sustained exosome release for over 14 days (Figure 1E), a feature attributable to its rational glucose‐responsive design.

**Figure 3:**
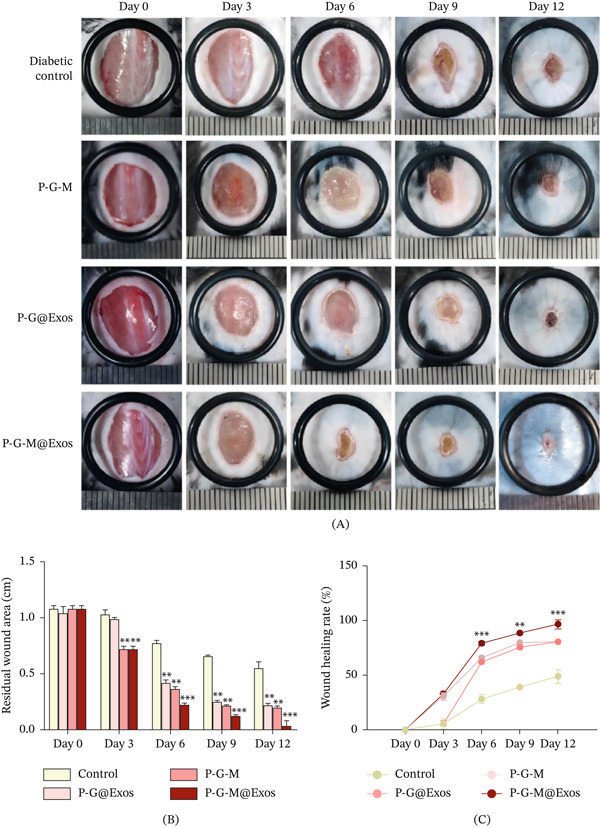
P‐G‐M@Exos hydrogel enhances diabetic wound healing in vivo. (A) Images representing the wound healing process in diabetic mice treated with PBS (diabetic control), P‐G‐M, P‐G@Exos, and P‐G‐M@Exos hydrogel (*n* = 5). (B) Comparison of residual wound area over time across various treatment groups and the control.  ^∗∗^ *p* < 0.01,  ^∗∗∗^ *p* < 0.001. (C) Evaluation of the rates of wound closure over time in various treatment groups compared with the control group.  ^∗∗^ *p* < 0.01,  ^∗∗∗^ *p* < 0.001.

In vivo assessments indicated that treatment with P‐G‐M@Exos significantly accelerated wound closure, achieving 77% by Day 6 (Figure 3A). After 12 days, wounds in the P‐G‐M@Exos group showed nearly complete closure (approximately 95%), compared with 75%–82% closure in groups treated with exosome‐free P‐G‐M or nonglucose‐responsive P‐G@Exos hydrogels, and only 42%–55% in the untreated control group (Figure 3B,C). The enhanced repair observed with P‐G‐M@Exos is likely attributable to the dual‐network architecture formed by PEGDA and GelMA, which improves mechanical stability and cellular adhesion [39, 40], in conjunction with the dynamic covalent boronate ester bonds provided by MPBA that enable glucose‐sensitive exosome release and enhanced bioavailability. The results underscore the promise of P‐G‐M@Exos as a viable treatment platform for chronic wound healing.

### 3.5. The P‐G‐M@Exos Hydrogel Supports Tissue Repair and Improves Collagen Deposition and Restructuring

**Figure 4:**
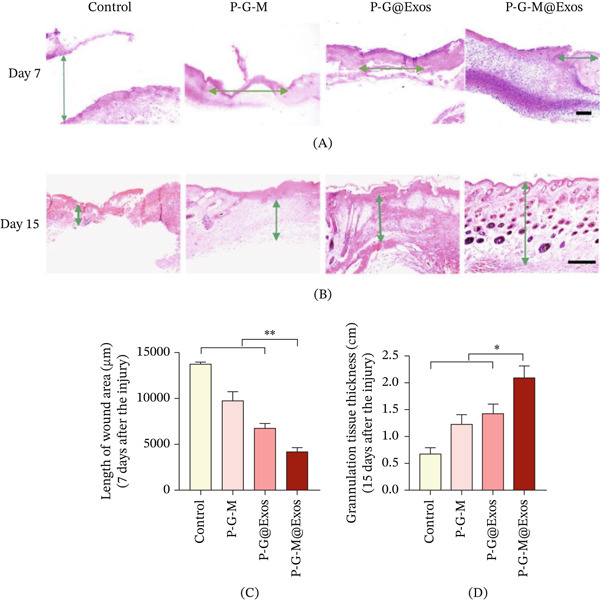
P‐G‐M@Exos hydrogel promotes tissue regeneration. (A) Representative H&E‐stained images of the wound area on Day 7 (*n* = 3). The wound area is indicated by green arrows to show its length. Scale bar: 500 *μ*m. (B) H&E‐stained wound sections illustrating granulation tissue formation on Day 15 (*n* = 3). Green arrows demarcate the granulation tissue thickness. Scale bar represents 250 *μ*m. (C) Quantitative analysis of the wound area length at Day 7.  ^∗∗^ *p* < 0.01. (D) Quantitative analysis of granulation tissue thickness at Day 15.  ^∗^ *p* < 0.05.

Histological evaluation was performed to assess the formation and maturation of granulation tissue (Figure 4A–D). By the seventh day postoperation, all hydrogel‐treated groups showed granulation tissue formation, whereas the control group continued to display incomplete neo‐epidermal coverage (Figure 4A). The P‐G‐M@Exos group exhibited the smallest wound gap, succeeded by the P‐G@Exos group, the exosome‐free P‐G‐M group, and finally the control group (Figure 4C). On Day 15, the granulation tissue thickness in the P‐G‐M@Exos group was significantly greater than that in all other groups (Figure 4B,D).

**Figure 5:**
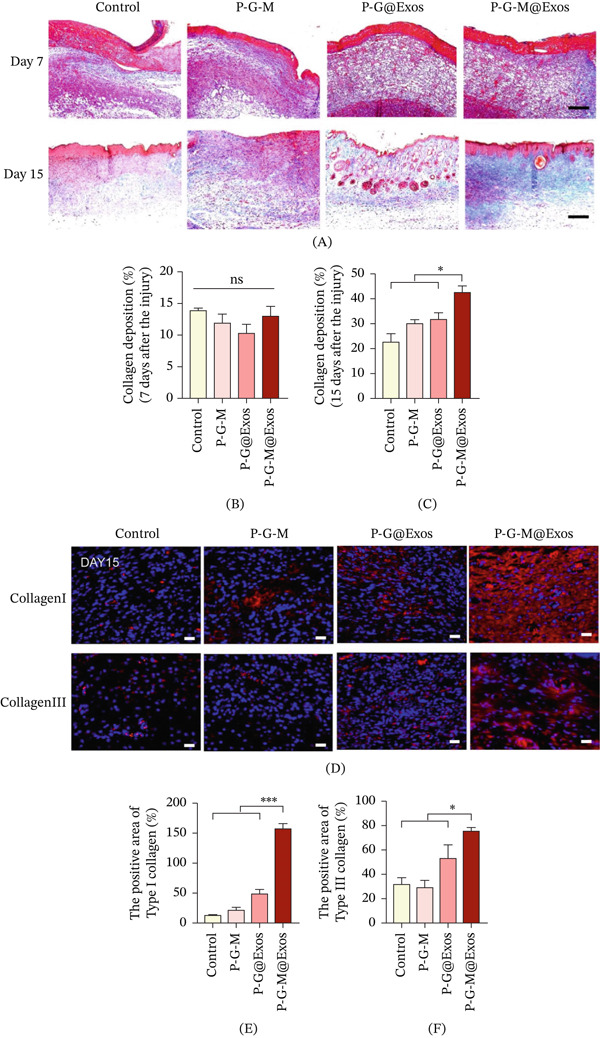
P‐G‐M@Exos hydrogel enhances collagen deposition and remodeling. (A) Collagen deposition in wound tissues is shown by Masson′s trichrome staining on Days 7 and 15 (*n* = 3). Scale bar, 250 *μ*m. (B, C) Quantitative analysis of collagen deposition using Masson′s trichrome staining was conducted on Days (B) 7 and (C) 15. ns indicates not significant;  ^∗^ *p* < 0.05. (D) Immunofluorescence staining on Day 15 reveals Type I and Type III collagen expression in wound tissue. Scale bar, 250 *μ*m. (E, F) Quantitative analysis of the immunofluorescence intensity for (E) Types I and (F) III collagen.  ^∗^ *p* < 0.05,  ^∗∗∗^ *p* < 0.001.

The accumulation and restructuring of collagen are essential for tissue repair during wound healing [41]. The collagen deposition observed on Day 7 using Masson′s trichrome staining was comparable between the P‐G‐M@Exos and control groups (Figure 5A,B). By the 15th day, however, the control group showed only minimal collagen deposition, whereas the P‐G‐M@Exos group exhibited abundant, aligned, and dense collagen fibers, indicating advanced tissue maturation (Figure 5A,C). Immunohistochemical analysis revealed that the P‐G‐M@Exos group exhibited significantly larger positive expression areas for Type I and Type III collagen compared with the control on Day 15, with a notably greater increase in Type I collagen observationally (Figure 5D–F). In accordance with classical wound healing dynamics, Type III collagen predominates in the early provisional matrix, whereas elevated expression of Type I collagen is indicative of later‐stage tissue remodeling [42]. These findings indicate that P‐G‐M@Exos not only promotes collagen synthesis but also directs the healing process toward a more mature and mechanically robust tissue architecture, ultimately enhancing the overall quality of wound regeneration.

### 3.6. Proangiogenic Effect of the P‐G‐M@Exos Hydrogel

**Figure 6:**
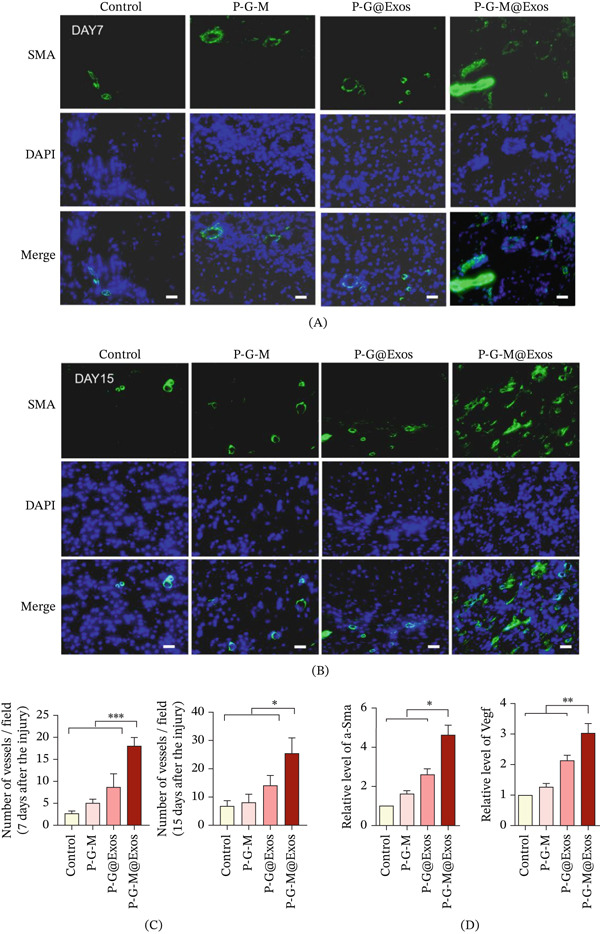
P‐G‐M@Exos hydrogel enhanced angiogenesis in vivo. (A–B) The wound bed′s blood vessels stained with *α*‐SMA (green) and DAPI (blue) are shown in representative immunofluorescence images for Days (A) 7 and (B) 15. Scale bar: 50 *μ*m. (C) The total count of blood vessels per field in the wound area was measured on Days 7 and 15 (*n* = 3). Significance is denoted by:  ^∗^ for *p* < 0.05 and  ^∗∗∗^ for *p* < 0.001. (D) On Day 7, qPCR was performed to evaluate the expression levels of *α-SMA* and *Vegf* genes (*n* =  3).  ^∗^ for *p* < 0.05 and  ^∗∗^ for *p* < 0.01.

Impaired wound healing in diabetic conditions is significantly influenced by dysfunctional angiogenesis. Effective vascularization can enhance re‐epithelialization and support efficient extracellular matrix remodeling [43, 44]. Immunofluorescence analysis showed a notable increase in *α*‐SMA‐positive cells within the granulation tissue of the P‐G‐M@Exos‐treated group versus the control group on Days 7 and 15, indicating a more mature and dense vascular network formation (Figure 6A–C). These observations were supported by qPCR analysis, which demonstrated a marked upregulation of angiogenesis‐related genes (*α-SMA and VEGF*) within the P‐G‐M@Exos group, with expression levels significantly surpassing those in both the P‐G@Exos and exosome‐free P‐G‐M groups (Figure 6D). These findings indicate that the P‐G‐M@Exos hydrogel effectively enhances angiogenesis in diabetic wounds at both molecular and tissue levels, providing essential microenvironmental support for improved healing.

### 3.7. P‐G‐M@Exos Hydrogel Promotes M2 Macrophage Polarization

**Figure 7:**
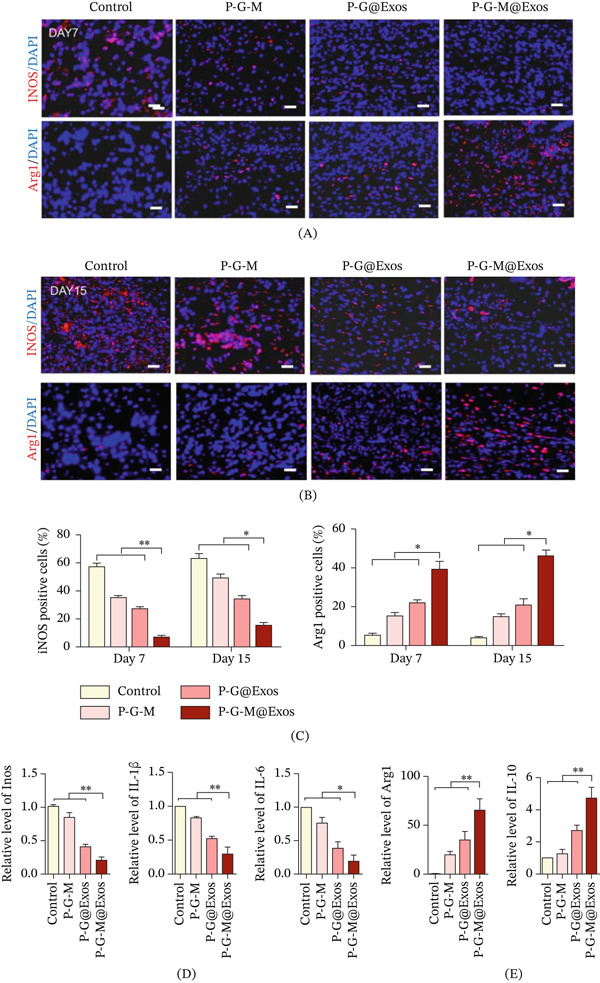
P‐G‐M@Exos hydrogel facilitates M2 macrophage polarization in diabetic wounds. (A–B) Representative immunofluorescence images of wound sections on Days 7 and 15 posttreatment, stained for Arg‐1^+^(red, M2 macrophage marker), iNOS^+^(red, M1 macrophage marker), and DAPI (blue, nuclear stain). Scale bar: 50 *μ*m. (C) Quantitative analysis of fluorescence intensity for Arg‐1 and iNOS expression in macrophages across various treatment groups.  ^∗^ *p* < 0.05,  ^∗∗^ *p* < 0.01. (D–E) On Day 7, qPCR analysis was conducted to evaluate the expression of genes associated with M1 macrophages (*iNOS, IL-1β, IL-6*) and M2 macrophages (*Arg-1, IL-10*).  ^∗^ *p* < 0.05,  ^∗∗^ *p* < 0.01.

Macrophages are crucial for immune responses and tissue repair. In diabetic wounds, the shift from proinflammatory M1 to proregenerative M2 macrophage phenotypes is often impaired, leading to a disrupted regenerative microenvironment and delayed healing [45]. The intrinsic antibacterial activity of the P‐G‐M hydrogel reduces early bacterial burden and dampens excessive M1‐polarizing stimuli (Figure 2A–D), thereby establishing a favorable microenvironment for macrophage phenotype switching. On this basis, the results indicated that treatment with P‐G‐M@Exos markedly boosted the presence of Arg1^+^ M2 macrophages—known for their immunomodulatory functions—on both Days 7 and 15. Conversely, the control group demonstrated a significant increase in iNOS^+^ M1 macrophages, known for their proinflammatory characteristics (Figure 7A–C). The study shows that P‐G‐M@Exos effectively combines antimicrobial properties with exosome‐mediated immunomodulation, shifting macrophage polarization from the proinflammatory M1 to the prorepair M2 phenotype, thus promoting tissue repair.

Chronic wounds are often marked by sustained inflammation, unlike acute wounds, where inflammation typically resolves within days [46]. In diabetic wounds, impaired resolution of inflammation further hinders the shift from M1 to M2 phenotypes [47]. To assess macrophage polarization, we examined the expression levels of genes linked to M1 and M2 phenotypes. Aligned with immunofluorescence findings, the P‐G‐M@Exos group exhibited the lowest M1‐related gene expression and the highest M2‐related marker expression compared with other groups (Figure 7D). The upregulation of *Vegf* aligns with the enhanced angiogenic capacity observed in this group (as shown in Figure 6D). In wound healing, M1 macrophages are primarily responsible for debridement and initiating inflammation, whereas M2 macrophages facilitate angiogenesis and matrix remodeling by secreting factors like VEGF in later stages [45]. These findings demonstrate that the P‐G‐M@Exos hydrogel synergizes with exosomes to modulate macrophage polarization, attenuate inflammatory responses, and significantly improve diabetic wound healing.

## 4. Conclusion

In summary, this research designed a unique multifunctional hydrogel based on a photo‐crosslinked P‐G‐M ternary‐network system. The hydrogel demonstrated tunable mechanical behavior, high hydrophilicity, favorable biocompatibility, and potent antibacterial activity. Critically, it enabled glucose‐responsive exosome release. In vivo, the exosome‐loaded P‐G‐M@Exos hydrogel significantly accelerated diabetic full‐thickness wound healing. This therapeutic effect was mediated by synergistic immunomodulation, notably through the induction of M2 macrophage polarization, coupled with enhanced angiogenic activity via *VEGF* upregulation. As the system is composed of biodegradable and biocompatible polymers PEGDA and GelMA, it provides a solid basis for future studies on its biodegradation and clinical application, highlighting its promise as an advanced dressing for diabetic wound care.

## Author Contributions

Conceptualization, methodology, formal analysis, writing—original draft: W.Y.; validation, data curation, investigation: Q.Z.; investigation: Q.L.; visualization: Z.L. and J.B.; software: Y.Z. and Y.Z.; supervision, resources: X.Y.; funding acquisition, project administration, writing—review and editing: J.Z.; W.Y. and Q.Z. have contributed to the work equally and should be regarded as co‐first authors.

## Funding

This study was funded by the Innovation Projects with Distinctive Features for Regular Higher Education Institutions in Guangdong Province (No.2023KQNCX030) and the Guangdong Province College Student Innovation Training Program (No.S202510573007).

## Disclosure

All authors have reviewed and approved the manuscript′s final version.

## Ethics Statement

The Institutional Animal Ethics Committee of Guangdong Pharmaceutical University approved the animal study protocol (Approval No. Gdpulac 2022141).

## Consent

The authors have nothing to report.

## Conflicts of Interest

The authors declare no conflicts of interest.

## Supporting information


**Supporting Information** Additional supporting information can be found online in the Supporting Information section. The following supporting information is available for download: Figure S1 (Characterization of hUC‐MSC‐Exos), Figure S2 (Comparison of normal wound and diabetic wound healing), and Table S1 (Primers applied in the qPCR examination).

## Data Availability

All relevant data are included within the manuscript and its Supporting Information.
