# Supplementary material for: Glucose‐Responsive PEGDA‐GelMA‐MPBA Hydrogel Loaded with Exosomes Promotes Diabetic Wound Healing
Source: J Diabetes Res. 2026 May 6;2026:3449648. doi: 10.1155/jdr/3449648 (PMC13150340; doi:10.1155/jdr/3449648)
Supplement: Supplementary file 1 — Supporting Information Additional supporting information can be found online in the Supporting Information section. The following supporting information is available for download: Figure S1 (Characterization of hUC‐MSC‐Exos), Figure S2 (Comparison of normal wound and diabetic wound healing), and Table S1 (Primers applied in the qPCR examination). [file JDR-2026-3449648-s001.docx]

**Supplementary Information**


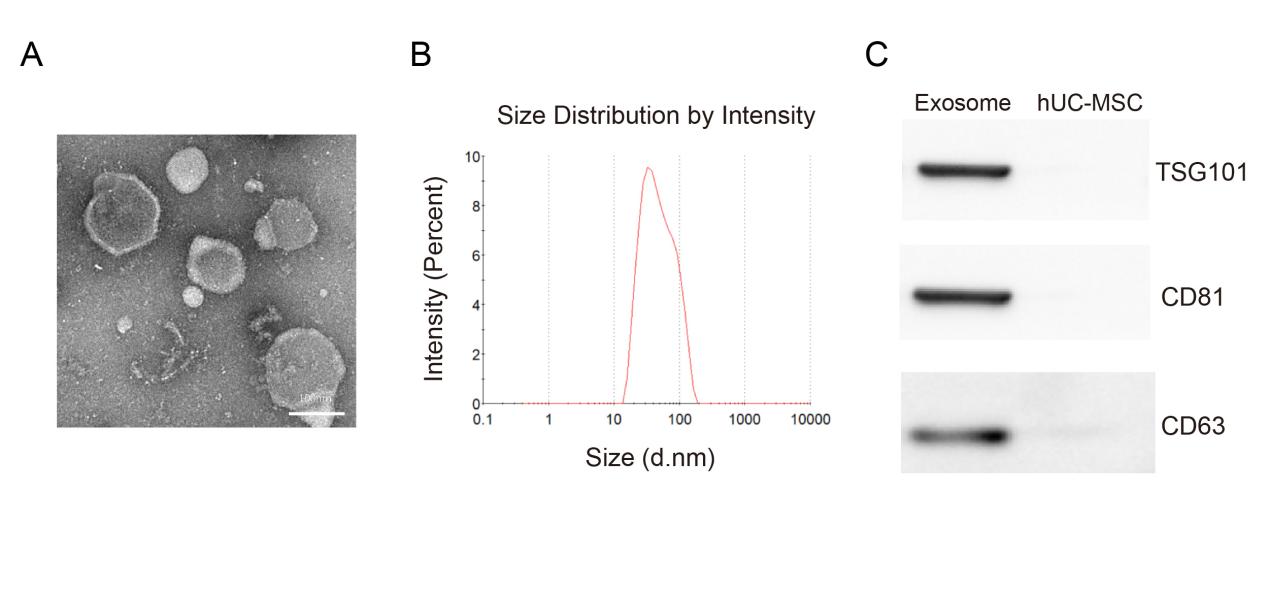


**Fig S1. Characterization of hUC-MSC-Exos.** (A) TEM imaging of hUC-MSC-Exos. Scale bar: 100 nm. (B) The size distribution detection of hUC-MSC-Exos is performed by NTA. (C) Western blot analysis of hUC-MSC-Exos markers of TSG101, CD81 and CD63.


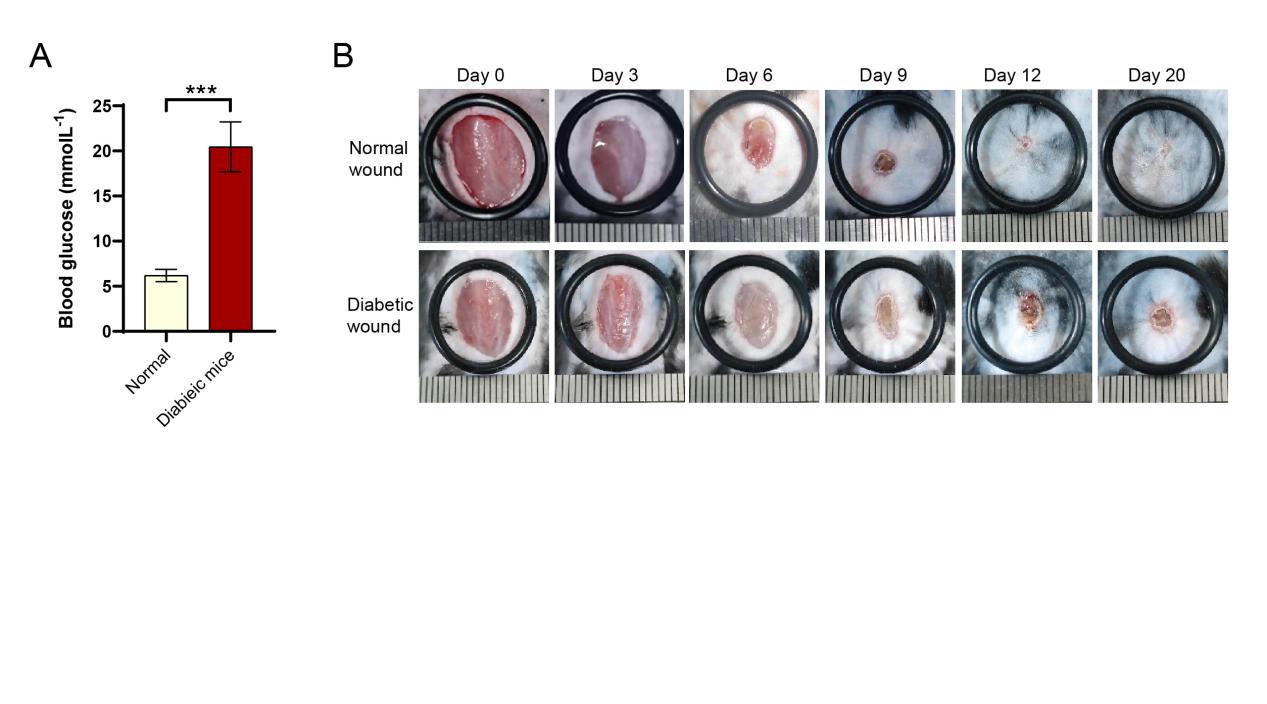


**Fig S2. Comparison of normal wound and diabetic wound healing.** (A) Identification of diabetic mice model (n = 14). ****P<0.001.* (B) Representative digital images of wound area in normal and diabetic mice at days 3, 6, 9, 12, and 20.

**Table**

**Table S1** Primers used in the qPCR analysis

| Genes | Forward primer | Reverse primer |
| --- | --- | --- |
| Actin | CATTGCTGACAGGATGCAGAAGG | TGCTGGAAGGTGGACAGTGAGG |
| α-SMA | GAGCGTGGCTATTCCTTCGTG | CAGTGGCCATCTCATTTTCAAAGT |
| Vegf | ACATTGGCTCACTTCCAGAAACAC | TGGTTGGAACCGGCATCTTTA |
| iNOS | GAGACAGGGAAGTCTGAAGCAC | CCAGCAGTAGTTGCTCCTCTTC |
| IL-6 | TTACACATGTTCTCTGGGA | GTTGTTCTTCATGTACTCCAG |
| IL-1β | GAAATGCCACCTTTTGACAGTG | TGGATGCTCTCATCAGGACAG |
| Arg-1 | AACACTCCCCTGACAACCA | CATCACCTTGCCAATCCC |
| IL-10 | CGGGAAGACAATAACTGCACCC | CGGTTAGCAGTATGTTGTCCAGC |
